# *Streptomyces hydrogenans* strain DH-16 alleviates negative impact of *Meloidogyne incognita* stress by modifying physio-biochemical attributes in *Solanum lycopersicum* plants

**DOI:** 10.1038/s41598-022-19636-0

**Published:** 2022-09-08

**Authors:** Nandni Sharma, Rajesh Kumari Manhas, Puja Ohri

**Affiliations:** 1 Department of Zoology, Guru Nanak Dev University, Amritsar, Punjab 143005 India; 2 Department of Microbiology, Guru Nanak Dev University, Amritsar, Punjab 143005 India

**Keywords:** Physiology, Plant sciences

## Abstract

The current study assessed the nematicidal and plant growth promoting potential of metabolites produced by *Streptomyces hydrogenans* strain DH-16 on morphological and physiological activities in 60 days old *Solanum lycopersicum* plants grown under *Meloidogyne incognita* stress. *M. incognita* infestation altered the levels of various photosynthetic pigments, various stress markers, enzymatic and non-enzymatic antioxidants in *S. lycopersicum* plants grown under in-vivo conditions. However, treatment with culture cells, supernatant and extract produced by *S. hydrogenans* strain DH-16 significantly reduced the number of galls in *M. incognita* infested plants when compared with untreated *M. incognita* infected plants. Moreover, the culture cells/ supernatant/ extract remarkably lowered the levels of stress markers (Hydrogen peroxide and Malondialdehyde) in infected plants and enhanced the activities of non-enzymatic antioxidants (glutathione, tocopherol) and enzymatic antioxidants (Catalase, Superoxide dismutase, Ascorbate peroxidase, Guaiacol peroxidase, Gluatathione-S-transferase and Polyphenol oxidase) in metabolites treated *M. incognita* infected plants. The enhanced level of different photosynthetic attributes were also evaluated by studying gas exchange parameters and different plant pigments. Moreover, an increment in the content of phenolic compounds such as total phenols, anthocyanin and flavonoids were also reflected in treated and nematode infested plants. The present study also evaluated the microscopic analysis depicting cell viability, nuclear damage and hydrogen peroxide localization in differently treated plants. The outcome of the present study therefore endorses the efficacy of DH-16 as a potential biocontrol agent that help plants in mitigating *M. incognita* stress.

## Introduction

*Solanum lycopersicum* (tomato) is one of the essential crops after potato that is cultivated worldwide because of its application in the agrofood industry^1^. The fruit of the plant is known to have high nutritional value, as it consists of antioxidants, flavonoids, vitamins, β-carotene, etc. But it is well documented that the growth and productivity of this crop is hindered by various abiotic and biotic stressors. Among biotic stressors, Plant parasitic nematode (PPN), *Meloidogyne incognita* acts as major constraint in the productivity of tomato crop, which further affect the maintenance of supply of this highly nutritious food item to the whole population, which is increasing at an alarming rate^2^.

*M. incognita* is a microscopic organism that resides in the soil and mainly infects the living tissues of host plant^3^. Second stage juveniles (J_2_) are the infective stage of *M. incognita* that penetrates into plant through roots and migrate towards the vascular tissue of plant^4^, afterwards moving towards the adjacent cells where they result in the formation of permanent feeding sites. Permanent feeding sites are basically formed of multicellular (giant) cells that further grow into root knot or galls^5^. These giant cells are the specific sites where *M. incognita* proliferates and develops an association with the host plant^4,6^. Moreover, PPNs also modulates the host protein machinery, thus resulting in protein denaturation and also alteration of various metabolic processes in host plants^7^. The main symptoms of PPNs infestation includes gall formation, disruptive water and mineral absorption and transportation, thus affecting the water-nutrient balance in plants, that further results in chlorosis and also makes the plant more susceptible to other pathogens^8,9^. In order to control the menace caused by these pathogens various nematode inhibitory chemicals are accessible from the market, but these chemical agents impose ill effects on non-target flora and fauna. Other strategies include crop rotation, use of resistant varieties, etc. but these strategies also exhibit certain limitations^2^. Despite all these methods, PPNs result in yearly loss of 100 billion dollars^10^. Thus, it is necessary to look for an effective environmentally safe management strategy for PPNs. One such strategy is the application of microbial organisms as biological control agents against PPNs^11^. Among microbes, Actinobacteria, which are G-C rich gram-positive bacteria are reported as the source of various biometabolites. Majority of these biometabolites include secondary metabolites in the form of agroactive compounds, for instance, Streptomycetes has been reported to act as biofertilizers that can increase fertility of soil and functional biodiversity in the rhizospheric region^12,13^. Furthermore, a number of plant growth promoting Actinomycetes also exhibits antifungal or antibacterial properties which was anticipated during their screening as biological control agents^14,15^.

Being rhizobacteria, Streptomycetes also promote plant growth, makes nutrients and minerals available to plants and also impose negative impact on different biotic stressors including *M. incognita* infestation^11,16,17^. Keeping into consideration the enhancing drift regarding the application of microorganisms as biological control agents, the current work has been framed for assessing the biocontrol and plant growth promoting potential of *S. hydrogenans* strain DH-16 against 60 days old nematode infested *S. lycopersicum* plants.

## Results

### Morphological parameters

**Figure 1:**
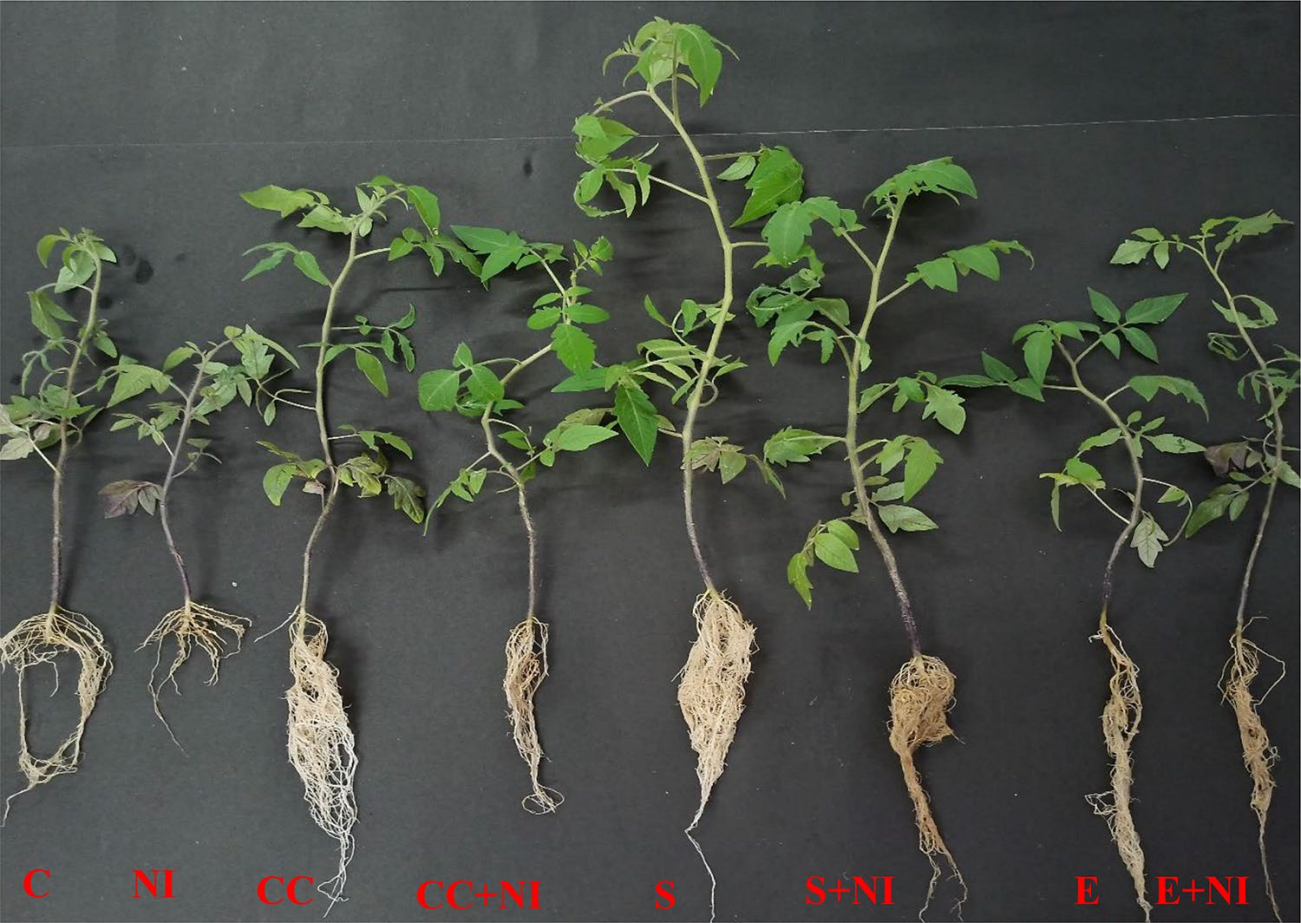
Figure depicting root length and shoot length of differently treated tomato plants. Different treatments include C (control); NI (nematode inoculated); CC (culture cells); CC + NI (culture cells + nematode inoculated); S (supernatant); S + NI (supernatant + nematode inoculated); E (extract); E + NI (extract + nematode inoculated).

The effect of biometabolites produced by *S. hydrogenans* strain DH-16 was examined on various morphological attributes of tomato plant like plant height (root, shoot length), fresh biomass (fresh root weight, fresh shoot weight) and nematode gall number on tomato roots (Fig. 1, Table 1). All these morphological parameters showed a considerable difference among different treatments in the current study. *M. incognita* infestation has resulted in decreased root length, shoot length and shoot weight by 38, 23 and 44%, respectively and the root weight was found enhanced in nematode infested plants by 17%, when compared with control plants. All these growth parameters were found enhanced in nematode infested plants treated with culture cells (CC), supernatant (S) and extract (E). Root length, shoot length, root and shoot weight was observed to be increased in culture cells (CC) treated plants by 99, 43, 138 and 166%, respectively when compared with untreated *M. incognita* stressed tomato plants. Moreover, amendment with Supernatant (S) also enhanced all these attributes by 147, 65, 567and 375% respectively, when compared with nematode infested untreated plants. Furthermore, treatment of extract (E) also enhanced all the parameters by 149, 28.7, 120 and 15% respectively, when compared with untreated nematode infected seedlings. But the increment of 8.25 and 10.3% was found in shoot length and shoot weight in case of culture cells treated nematode infected plants when assessed against their counter control plants (i.e., uninfected culture cells treated plants). However, culture cell treated nematode inoculated plants showed decrease in root length and root weight by 21.83 and 42.9% respectively, when compared to uninfected culture cell amended tomato plants. Further all these respective growth attributes except for gall number were found enhanced in nematode infected supernatant treated tomato by 9.3, 2.4, 11.27 and 5.3% respectively, when compared to their counter control plants. Similarly, root and shoot length were found enhanced in nematode infected extract treated plants by 10.16 and 2.15%, but root weight and shoot weight were found decreased by 24 and 36% respectively, when compared to uninfected extract amended tomato plants.

**Table 1:** Morphological parameters of *M. incognita* inoculated 60 days old tomato plants treated with *S. hydrogenans* strain DH-16.

Treatments	Morphological parameters (Mean ± S.E)				
	Root length (cm)	Shoot length (cm)	Fresh root weight (g)	Fresh shoot weight (g)	Number of galls
C	14.00 ± 1.53^ab^	31.67 ± 0.88^abc^	0.29 ± 0.049^a^	2.23 ± 0.64^a^	–
NI	8.67 ± 1.09^a^	24.33 ± 2.40^a^	0.34 ± 0.046^a^	1.24 ± 0.27^a^	26 ± 5.81^b^
CC	22.17 ± 2.18^c^	32.33 ± 1.33^abc^	1.42 ± 0.38^ab^	2.99 ± 0.36^ab^	–
CC + NI	17.33 ± 1.48^bc^	35.00 ± 1.15^bc^	0.81 ± 0.061^ab^	3.30 ± 0.12^abc^	11 ± 0.33^a^
S	19.67 ± 1.20^bc^	41.33 ± 1.20^c^	2.04 ± 0.63^ab^	5.59 ± 0.52^bc^	–
S + NI	21.5 ± 1.26^c^	40.33 ± 3.71^bc^	2.27 ± 0.67^b^	5.89 ± 1.24^c^	15 ± 2.31^ab^
E	19.67 ± 1.09^bc^	30.67 ± 1.20^ab^	0.99 ± 0.24^ab^	2.24 ± 0.13^a^	–
E + NI	21.67 ± 1.69^c^	31.33 ± 3.28^abc^	0.75 ± 0.056^ab^	1.43 ± 0.21^a^	10 ± 2.40^a^
F-value	9.887**	6.518**	4.129**	9.975**	4.967*

Different treatments are represented as C (control), NI (nematode inoculated), CC (culture cells), CC + NI (culture cells + nematode inoculated), S (supernatant), S + NI (supernatant + nematode inoculated), E (crude extract), E + NI (crude extract + nematode inoculated). Results are given in mean ± standard error and F-value * depicts significant difference at *p* ≤ 0.05.

** indicates significant difference at *p* ≤ 0.01 as per Tukey’s test. Letters a, b, c depicts significant difference among treatments and same letters depicts no significant difference.

The number of nematode galls on plant roots were significantly reduced in culture cells, supernatant and extract treated plants by 57.6, 42.3 and 61.5%, respectively when compared to non-treated infected plants.

### Photosynthetic pigments

**Figure 2:**
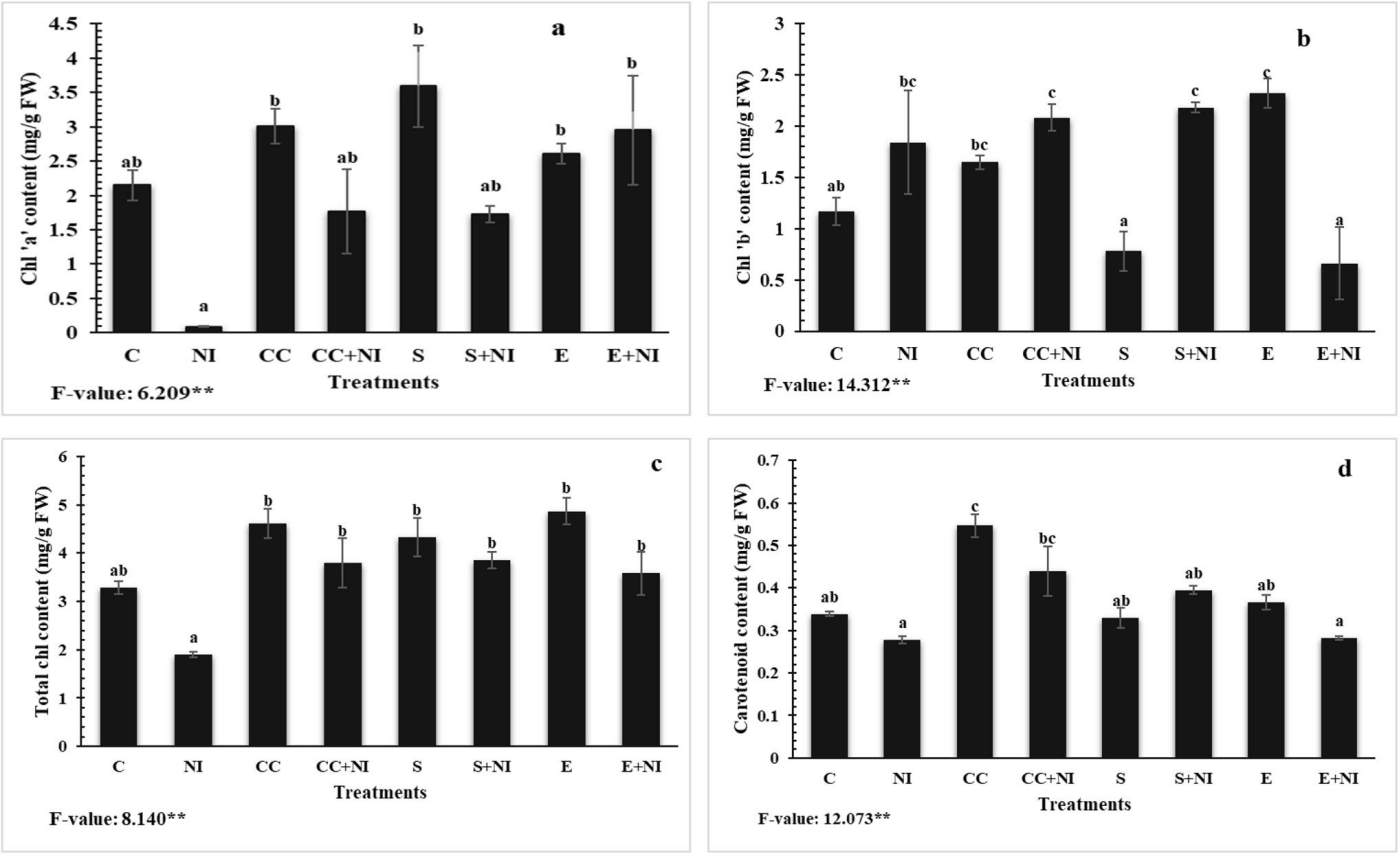
(**a-d**) Photosynthetic pigments (**a**) Chlorophyll ‘a’ content; (**b**) Chlorophyll ‘b’ content; (**c**) Total chlorophyll content; (**d**) Carotenoid content in *M. incognita* inoculated 60 days old tomato plants treated with *S. hydrogenans* strain DH-16. Different treatments are represented as C (control), NI (nematode inoculated), CC (culture cells), CC + NI (culture cells + nematode inoculated), S (supernatant), S + NI (supernatant + nematode inoculated), E (crude extract), E + NI (crude extract + nematode inoculated). Results are given in mean ± standard error and F-value ** indicates significance at *p* ≤ 0.01 as per Tukey’s test. Different letters a, b, c represents significant difference among treatments and same letters represent no significant difference.

The levels of Chl ‘a’, total Chl and carotenoid content were significantly reduced in nematode infested plants by 95.86, 42 and 17.7% respectively, when compared to control plants. The levels of chl ‘a’, chl ‘b’,total chl and carotenoid content in culture cells treated nematode infected tomato plants were elevated by 188.3, 13.17, 100.3 and 57.9% respectively when compared to untreated nematode infested plants. Moreover, the levels were also enhanced in supernatant treated plants by 1835, 18.4, 103 and 41.6% respectively, when compared to non-treated nematode infested tomato plants. The levels of photosynthetic pigments were also significantly enhanced in extract treated nematode infested tomato plants by 3215, 64, 88.8 and 1.29% respectively, when compared to untreated nematode infested tomato plants. However, all these parameters except chl ‘b’ were found down- regulated in culture cells treated nematode infected plants by 41.39, 17.55 and 19.5% respectively when compared to uninfected culture cells amended plants. Similarly, chl ‘a’ and total chl were found decreased in supernatant amended nematode inoculated plants by 52.03 and 11.08%, when compared to uninfected supernatant amended plants. Moreover, in case of extract amended nematode inoculated plants, decline in the levels of total chl and carotenoid content was observed by 26.38 and 23.11% respectively, when compared to uninfected extract amended tomato plants (Fig. 2a-d, Table S1).

### Gas analysis parameters

**Figure 3:**
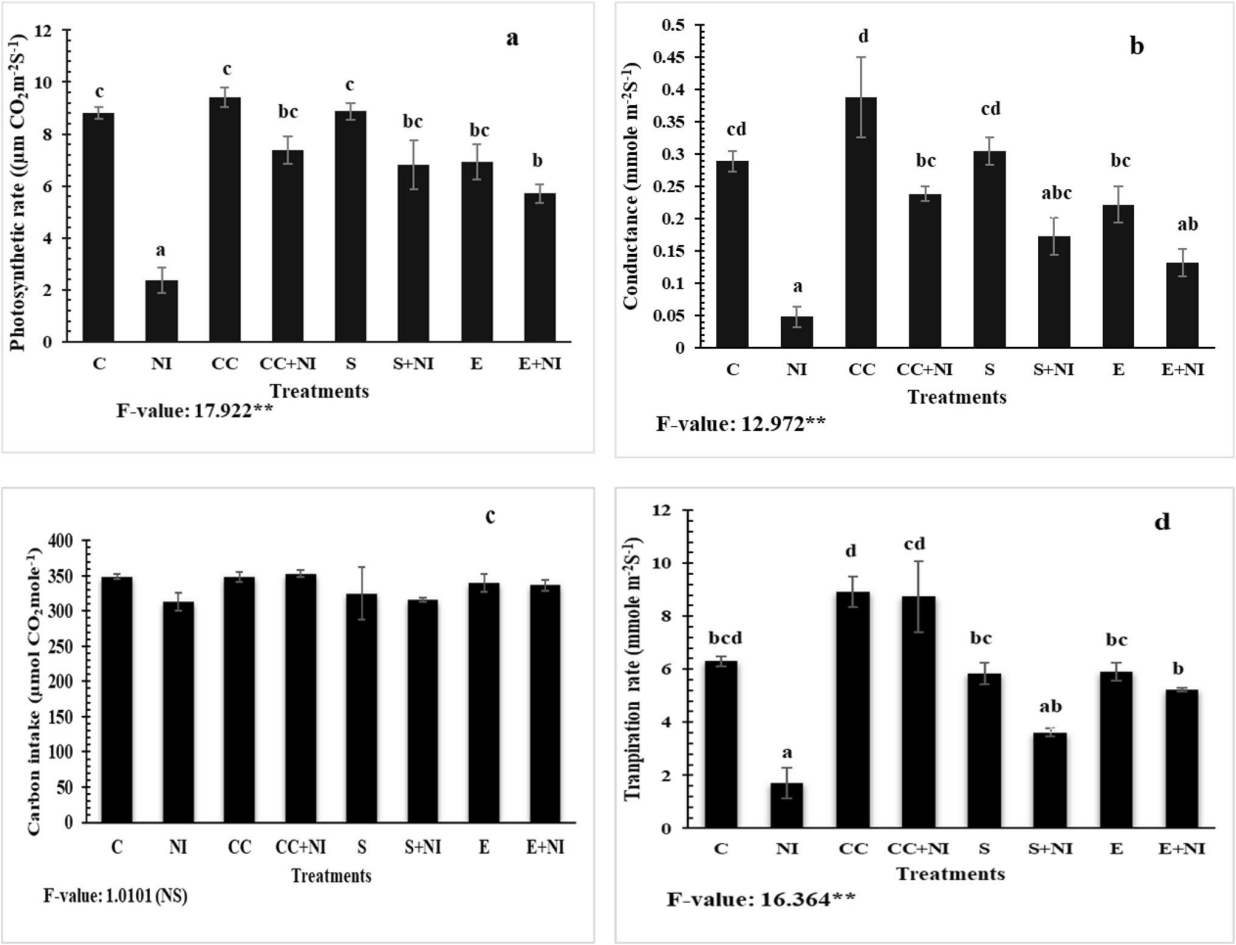
(**a-d**) Gas analysis parameters (**a**) Photosynthetic rate; (**b**) Conductance; (**c**) Carbon intake; (**d**) Transpiration rate in *M. incognita* inoculated 60 days old tomato plants under treatment with *S. hydrogenans* strain DH-16. Different treatments are represented as C (control), NI (nematode inoculated), CC (culture cells), CC + NI (culture cells + nematode inoculated), S (supernatant), S + NI (supernatant + nematode inoculated), E (crude extract), E + NI (crude extract + nematode inoculated). Results are given in mean ± standard error and F-value ** indicates significance at *p* ≤ 0.01 and ns indicates non-significant difference as per Tukey’s test. Different letters a, b, c, d represent significant difference among treatments and same letters represent no significant difference.

Various gas analysis parameters like photosynthetic rate, conductance, carbon intake and transpiration rate were found decreased in nematode infested plants by 73, 83, 10.3 and 72.7% respectively, when compared to control plants. But the levels were enhanced in plants treated with culture cells by 212, 400, 12.7 and 408% respectively, when compared to untreated nematode infected plants. Furthermore, treatment with supernatant also elevated the levels of photosynthetic rate, conductance, carbon intake and transpiration rate by 188, 262, 0.95 and 110% respectively, when compared to untreated nematode infested plants. The treatment of extract also enhanced the levels of all the parameters by 141, 175, 7.6 and 204% respectively when compared to untreated *M. incognita* infested plants (Fig. 3a-d). Similarly, all the gas exchange parameters except carbon intake were found decreased in case of culture cell treated nematode infected plants by 21.5, 38.47 and 2.01% respectively, when evaluated against uninfected culture cells amended plants. However, decline of 23.06, 43.37, 2.76 and 38.21% in all these attributes was reported in nematode infested supernatant amended tomato plants, when compared to uninfected supernatant supplemented tomato plants. Similar decline of 17.35, 40.7, 0.88 and 11.50% was observed in nematode infected extract treated plants, when compared to uninfected extract supplemented plants (Fig. 3a-d; Table S2).

### Oxidative burst

**Figure 4:**
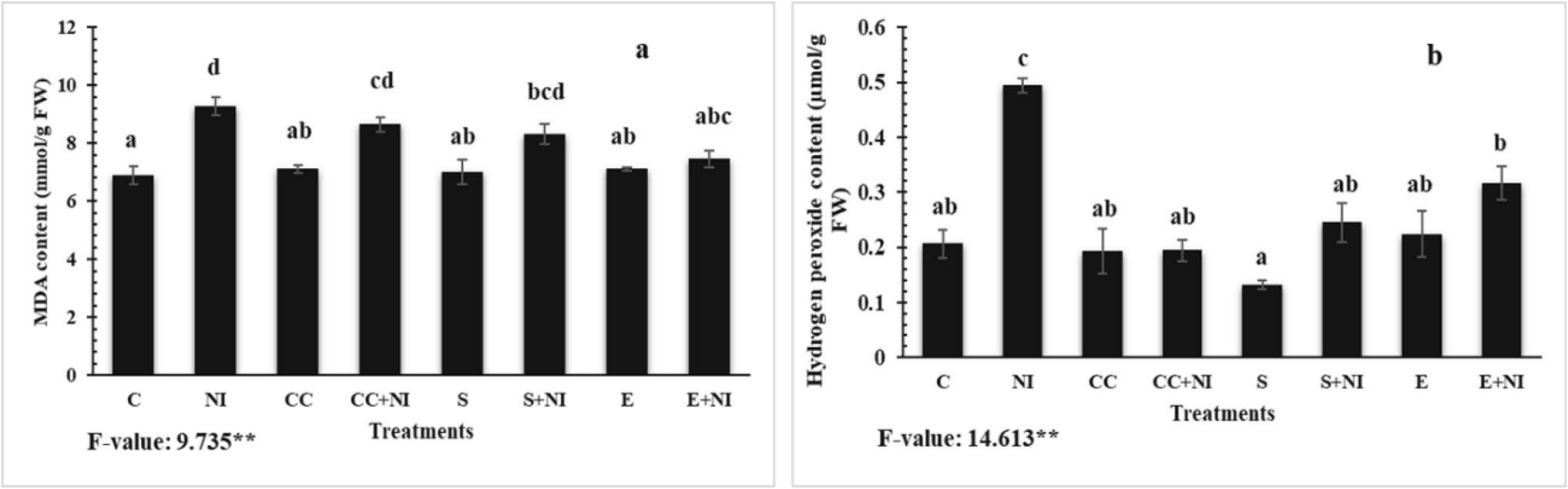
(**a-b**) Level of stress markers (**a**) MDA content; (**b**) H_2_O_2_ content in *M. incognita* inoculated 60 days old tomato plants treated with bioactive components produced by *S. hydrogenans* strain DH-16. Different treatments are represented as C (control), NI (nematode inoculated), CC (culture cells), CC + NI (culture cells + nematode inoculated), S (supernatant), S + NI (supernatant + nematode inoculated), E (crude extract), E + NI (crude extract + nematode inoculated). Results are given in mean ± standard error form and F-value ** indicates significance at p ≤ 0.01 as per Tukey’s test. Different letters a, b, c, d represent significant difference among treatments and same letters represent no significant difference.

The level of MDA and H_2_O_2_ were found enhanced in nematode infested seedlings by 34 and 138.9%, respectively, when compared to untreated and uninfected control plants. The level was further elevated in culture cells treated nematode infested plants by 6.8 and 61.26%, respectively when compared with nematode infested untreated plants. Furthermore, amendment of nematode infected tomato plant with supernatant also resulted in enhanced levels of MDA and H_2_O_2_ by 10.5 and 50.4%, respectively. The level was also elevated in nematode infested extract (E) treated plants by 19.7 and 35.8%, when compared to nematode infested untreated tomato plants. Further increment of 21.82 and 0.36% in the levels of MDA and H_2_O_2_ was observed in case of nematode infected culture cells treated tomato plants, when compared to their respective counter control plants. A similar increase of 18.45 and 85.73% was observed in supernatant treated nematode infected plants, when compared to uninfected supernatant supplemented plants. A similar trend of enhancement by 4.65 and 4.36% was also seen in case of nematode infected extract treated plants when compared to uninfected extract amended tomato plants(Fig. 4a,b; Table S3).

### Antioxidative enzymes

The efficacy of biometabolites was also assessed in nematode infested plants by studying the specific activities of various antioxidative enzymes. The level of various enzymes like CAT, SOD, APOX, GuPOX, PPO and GST was found elevated in nematode infested tomato plants by 157, 191, 235, 132, 46 and 75%, respectively when compared to the control plants. The level of these enzymes were further enhanced in culture cells treated *M. incognita* infested plants by 29, 11, 34, 9.6, 9.4 and 28.6% respectively, when compared with *M. incognita* infested untreated tomato plants. Amendment of supernatant also elevated the activities of CAT, SOD, APOX, PPO and GST by 19, 91.4, 63.8, 20.4 and 11.8% respectively when compared with untreated nematode infected plants. Furthermore, treatment of extract also resulted in increment in the activities of CAT, SOD, APOX, POD, PPO and GST by 26, 91.4, 24.2, 61.9, 65 and 34.56% respectively when compared to untreated nematode infested plants. The specific activity of all these enzymes were found enhanced in culture cells treated nematode infected plants by 210, 69.56, 79.77, 73, 73.4 and 65% respectively, when assessed against uninfected culture cells treated plants. Similarly, the activities of all these enzymes except SOD were found upregulated by 160, 56.5, 7.118, 48.6 and 168.6% respectively, when compared to their counter control plants. Further, the specific activity of all these enzymes except SOD and GST were found enhanced by 85.71, 77, 38.59 and 16.89% respectively in case of extract amended nematode inoculated plants, when evaluated against uninfected extract supplemented plants (Table 2).

**Table 2:** Specific activities of antioxidative enzymes in *M. incognita* inoculated 60 days old tomato plants treated with *S. hydrogenans* strain DH-16.

Treatments	Specific activity (Mean ± S.E.)					
	Catalase (U/mg protein)	Superoxide dismutase (U/mg protein)	Ascorbate peroxidase (U/mg protein)	Guaiacol peroxidase (U/mg protein)	Polyphenol oxidase (U/mg protein)	Glutathione-s-transferase (U/mg protein)
C	0.0028 ± 0.00024^a^	0.0012 ± 0.00095^a^	0.0422 ± 0.00940^a^	0.8152 ± 0.05150^a^	0.2007 ± 0.01712^a^	0.2120 ± 0.01188^a^
NI	0.0072 ± 0.00008^bc^	0.0035 ± 0.00069^a^	0.1415 ± 0.01660^bc^	1.8973 ± 0.18550^b^	0.2933 ± 0.00800^ab^	0.3718 ± 0.00447^bc^
CC	0.0030 ± 0.00005^a^	0.0023 ± 0.00019^a^	0.1058 ± 0.03220^abc^	1.2017 ± 0.16405^ab^	0.1851 ± 0.02440^a^	0.2883 ± 0.01700^ab^
CC + NI	0.0093 ± 0.00033^c^	0.0039 ± 0.00011^ab^	0.1902 ± 0.00700^ cd^	2.0802 ± 0.07866^bc^	0.3211 ± 0.05796^abc^	0.4784 ± 0.01386^ cd^
S	0.0033 ± 0.00057^a^	0.0084 ± 0.00087^c^	0.1481 ± 0.01274^bcd^	1.2755 ± 0.22196^ab^	0.2375 ± 0.02435^ab^	0.1548 ± 0.02393^a^
S + NI	0.0086 ± 0.00011^c^	0.0067 ± 0.00045^bc^	0.2318 ± 0.02170^d^	1.3663 ± 0.16955^ab^	0.3531 ± 0.03491^abc^	0.4158 ± 0.01625^bcd^
E	0.0049 ± 0.00111^ab^	0.0069 ± 0.00074^bc^	0.0993 ± 0.01906^ab^	2.2171 ± 0.38268^bc^	0.4148 ± 0.07108^bc^	0.5185 ± 0.07099^d^
E + NI	0.0091 ± 0.00021^c^	0.0067 ± 0.00048^bc^	0.1758 ± 0.00503^bcd^	3.0727 ± 0.22736^c^	0.4849 ± 0.00835^c^	0.5003 ± 0.01782^ cd^
F-value	36.090**	16.406**	11.399**	12.028**	7.789**	21.866**

Different treatments are represented as C (control), NI (nematode inoculated), CC (culture cells), CC + NI (culture cells + nematode inoculated), S (supernatant), S + NI (supernatant + nematode inoculated), E (crude extract), E + NI (crude extract + nematode inoculated). Results are given in mean ± standard error and F-value ** indicates significance at *p* ≤ 0.01 as per Tukey’s test. Different letters a, b, c, d represent significant difference among treatments and same letters represent no significant difference.

### Non-enzymatic antioxidants

**Figure 5:**
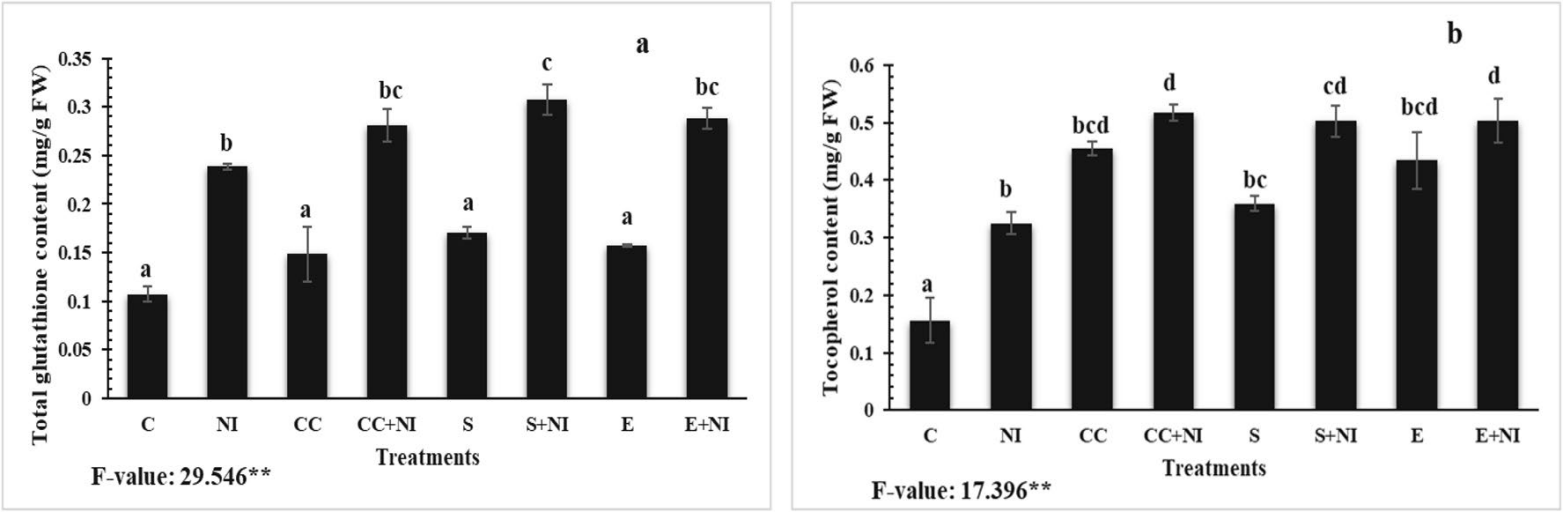
(**a-b**) Levels of non-enzymatic antioxidants (**a**) Total glutathione content; (**b**) Tocopherol content in *M. incognita* inoculated 60 days old tomato plants treated with *S. hydrogenans* strain DH-16 in the form of culture cells (CC), supernatant (S) and crude extract (E). Different treatments are represented as C (control), NI (nematode inoculated), CC (culture cells), CC + NI (culture cells + nematode inoculated), S (supernatant), S + NI (supernatant + nematode inoculated), E (crude extract), E + NI (crude extract + nematode inoculated). Results are given in mean ± standard error and F-value ** indicates significance at *p* ≤ 0.01 as per Tukey’s test. Different letters a, b, c, d represent significant difference among treatments and same letters represent no significant difference.

Total glutathione and tocopherol contents were also assessed and were found enhanced in plants growing under the influence of nematodes by 122 and 108% when compared to control plants. The levels were found enhanced further in culture cells treated nematode infested plants by 17.89 and 59.08% respectively, when compared with nematode infected untreated plants. Moreover, application of supernatant also enhanced the levels of glutathione and tocopherol by 28.79 and 54.58% respectively, when evaluated against nematode inoculated untreated plants. The levels were also enhanced in extract treated plants by 20.8 and 55.11% respectively, when compared to nematode infested untreated tomato plants. However, an enhancement in total glutathione and tocopherol content by 89.3 and 13.74% was reported in case of culture cells treated nematode inoculated plants, when compared to uninfected culture cells treated plants. Similar upregulation of 89.3 and 13,74% respectively was observed in supernatant treated nematode inoculated plants, when compared to uninfected supernatant treated plants. Additionally extract amended nematode infected plants also showed an increment of 83.28 and 16.13% repectively, when compared to their counter control plants (Fig. 5a-b; Table S4).

### Phenolic compounds

**Figure 6:**
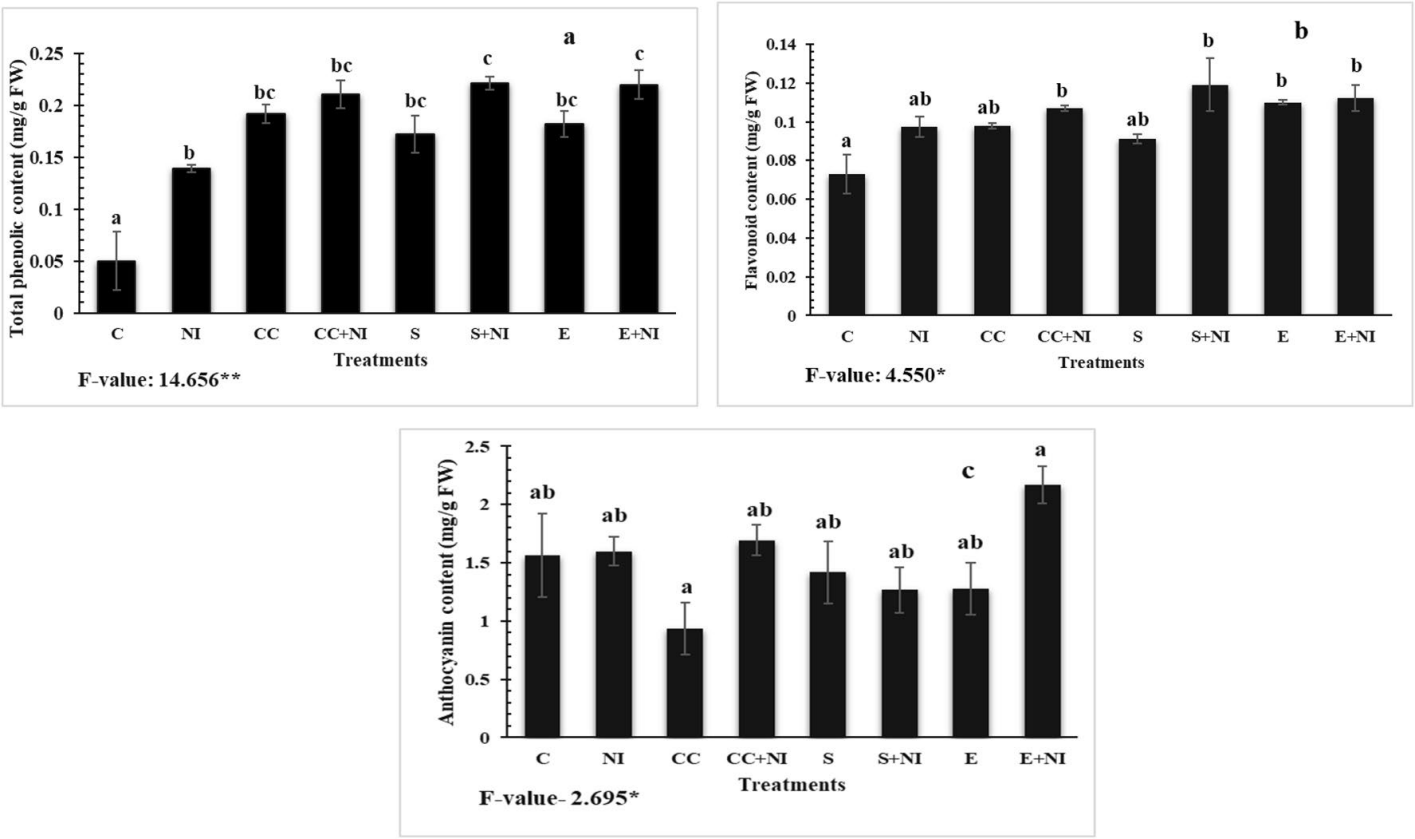
(**a-c**) Level of phenolic compounds (**a**) Total phenolic content; (**b**) Flavonoid content; (**c**) Anthocyanin content in *M. incognita* inoculated 60 days old tomato plants treated with *S. hydrogenans* strain DH-16. Different treatments are represented as C (control), NI (nematode inoculated), CC (culture cells), CC + NI (culture cells + nematode inoculated), S (supernatant), S + NI (supernatant + nematode inoculated), E (crude extract), E + NI (crude extract + nematode inoculated). Results are given in mean ± standard error and F-value *indicates significance at p ≤ 0.05 as per Tukey’s test. Different letters a, b represents significant difference among treatments and same letters represent no significant difference.

Total phenolic, flavonoid and anthocyanin contents were found increased in nematode infested plants by 179, 35 and 22% respectively, when compared to control plants. But the levels were enhanced in culture cells treated nematode infested plants by 51, 10 and 6% respectively, when compared to untreated nematode infected plants. Furthermore, amendment with supernatant also enhanced the levels by 59, 22 and 36% respectively, when compared to nematode infected untreated plants. The treatment of extract also elevated the levels of phenolic and flavonoid compounds by 58 and 15% respectively, when compared with untreated nematode infected plants. However, an increment of 81.3% was observed in the level of anthocyanin in culture cells treated nematode infected tomato plants, when compared to uninfected culture cells treated plants. Similar, upregulation of 70.23% was also observed in nematode infected extract supplemented plants, when assessed against extract treated control tomato plants (Fig. 6a-c; Table S5).

### Microscopic studies

**Figure 7:**
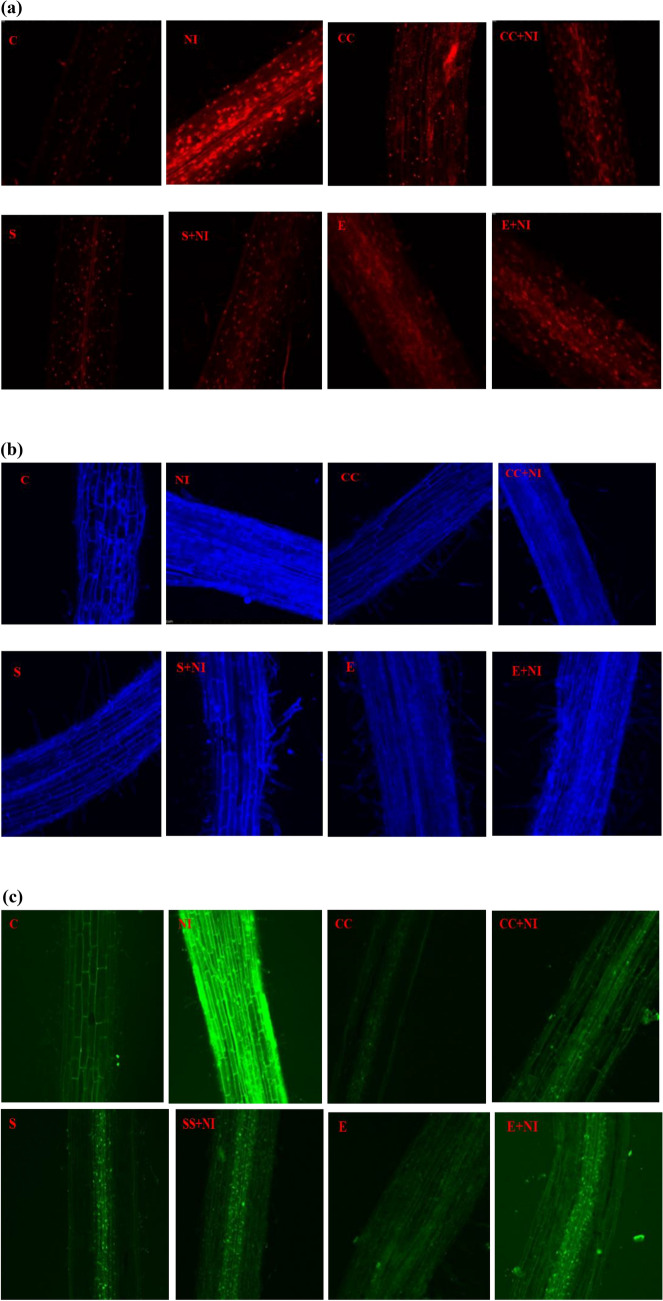
(**a–c**) Micrographs depicting (**a**) cell viability; (**b**) membrane damage; (**c**) hydrogen peroxide localization in differently treated *S. lycopersicum* seedlings. Different treatments are represented as C (control), NI (nematode inoculated); CC (culture cells); CC + NI (culture cells + nematode inoculated); S (supernatant); S + NI (supernatant + nematode inoculated); E (extract); E + NI (extract + nematode inoculated).

Cell viability was evaluated on the basis of intensity of red colour. It was observed that the maximum damage, as indicated by intensity of Propidium iodide (PI) was seen in *M. incognita* infected plants when compared to control tomato plants, indicating that the uninfected control plants were more viable. Furthermore, treated nematode infected plants were less damaged as compared to untreated nematode inoculated plants (Fig. 7a).

Nuclear damage was also assessed on the basis of the intensity of blue colour, which was found to be maximum in case of untreated nematode infected plants when compared to control and metabolites supplemented nematode infected plants (Fig. 7b).

Hydrogen peroxide tagging was also studied as per the intensity of green colour (because of DCF-DA) and the maximum tagging was found in nematode infected plants in comparison to control plants, depicting maximum oxidative stress in nematode stressed plants. Moreover, the tagging was less in case of biometabolites supplemented nematode infected tomato plants (Fig. 7c).

## Discussion

Since, *M. incognita* imposes a negative impact on the yield and productivity of almost all the crops, various biocontrol agents are being explored for the management of PPNs. Moreover, various factors like environmentally safe nature, low-cost value and easy mode of application make the use of biocontrol microbes a more efficient approach for controlling PPNs population^18,19^. Therefore, in present study we have evaluated the potential of biometabolites produced by *S. hydrogenans* in the form of culture cells, supernatant and extract against *M. incognita* infecting *S. lycopersicum* plants growing under in-vivo conditions.

In the present work, it has been found that *M. incognita* infestation results in reduced growth attributes (root length, shoot length, shoot weight) in 60 days old *S. lycopersicum* plants. This reduction is attributed to the disruption in the structure of root in the form of galls, which further alters the uptake and transportation of water and nutrients from the roots to other parts of the host plant, thus ultimately imposing a negative impact on normal metabolism in plants. However, the root weight was found enhanced in nematode infected plants when compared to healthy control plants, this enhancement might be because of heavily infected roots (with large number of galls). Our findings are in agreement with the study of Abd-El-Al and co-workers^20^ in case of *Luffa aegyptiaca* (Sponge gourd). Additionally, the similar trend of reduction in growth parameters was also reported in case of *M. incognita* infected black gram plants^21^ and galls were formed in nematode infected plants. Gall formation occurs because of the rapid multiplication of nematodes after penetrating the root tissue of host plants. Similar findings were also reported in case of *Matricaria reculita* and *S. lycopersicum* plants^22,23^.

It has been noticed that amendment of biometabolites produced by microbial strain enhanced the root length, shoot length, root weight and shoot weight in *M. incognita* infested *S. lycopersicum* plants. In addition to growth attributes, the reduction in the number of galls was also found in treated plants. In previous studies also, it was observed that biometabolites produced by *Streptomyces* sp. promoted all the growth parameters and decreased the number of galls in nematode stressed tomato plants^23^. Our results also coincide with the findings of Mekete and co-workers^24^, where reduction in gall number was observed in case of *Bacillus* sp. supplemented Ethiopian coffee plants. These results might be because of the production of nematicidal compounds by microbial strains, which results in reduced gall formation and thus promotes the growth of the host plant.

The results derived from the current work suggested that nematode infestation also reduced the level of photosynthetic pigments like chl ‘a’, chl ‘b’, total chlorophyll and carotenoid in 60 days old tomato plants. These results concur with the previous studies in case of tomato and capsicum plants infected with nematodes^23,25^. This reduction in pigments is attributed to the formation of galls over the surface of the root, which further disrupts the transportation of water and also the level of photosynthetic pigments^26^. Our finding also depicts significant elevation in the level of plant pigments in case of biometabolites treated nematode infested tomato plants. These results are in agreement with the findings of Abd-El-Khair and co-workers^27^ which states that supplementation of *B. subtilis*, *B. pumilus* and *P. fluoriscens* promoted the content of pigments in nematode infected cowpea plants. Moreover, Sharma and group^28^ also reported an upregulation in the level of these pigments in *S. antibioticus* treated tomato plants that were growing under nematode stress. This increment in the level of pigments might be because of the role of microbes in promoting translocation of minerals and also in safeguarding photosynthetic machinery.

Downregulation in various gaseous exchange parameters has also been reported in *M. incognita* infected *S. lycopersicum* plants. Similar reports were reported previously in the case of coffee and tomato plants infected with nematodes^29,30^. This downregulation is attributed to hindrance in water uptake because of gall formation^31^. Our study also reveals that amendment of plants with biometabolites produced by *S. hydrogenans* results in elevation of all the gas exchange parameters. These results corroborate with the outcomes of Khanna et al.^30^ in case of *M. incognita* infested microbial strain treated 45 days old tomato plants. The reason behind this increment might be the upregulation in the synthesis of enzymes involved in violaxanthin pathway (photosynthetic processes).

An elevation in the content of various stress markers or ROS like MDA and H_2_O_2_ has been reported in nematode infected plants in present study. Our results find support from the previous studies of Sharma and co-workers^23^, where MDA and H_2_O_2_ were reported to enhance in tomato plants grown under nematode stress. Similar trend of increase in MDA was also reported in the case of nematode inoculated sugar beet plants^32^. However, the current study also depicts the downregulation in the level of stress markers in biometabolites amended nematode infected tomato plants. Similar decline was also reported in nematode infested *Baccopa monnieri* plants when treated with *Chitinphilus* sp. and *Streptomyces* sp. This down regulation is attributed to the generation of immunity-based cascade by microorganisms so as to mitigate pathogenesis in plants^33^. Another possible reason behind this down regulation might be the enhancement in the activity of various enzymatic and non-enzymatic antioxidants^11^. Earlier reports of Khanna et al.^34^ also assessed decline in lipid peroxidation and H_2_O_2_ in *P. aeruginosa* and *B. gladioli* treated nematode infected tomato plants. It was predicted that the decline in stress markers is due to microbe induced enhanced activity of the antioxidant defense system of plants. Furthermore, microorganisms were also reported to produce certain secondary metabolites like siderophores and organic acid that acts as chelators of ROS in plants.

The current work also evaluates an outburst in the specific activity of various antioxidant defense enzymes (CAT, SOD, APOX, POD, PPO and GST) in nematode infected tomato plants. Our results find support from the previous work done on tomato plants, where activity of all the enzymes were enhanced, further reducing the level of ROS that was upregulated in plants because of nematode pathogenesis^11^. Additionally, enhanced specific activity of SOD, PPO and POD were also reported in *Gerbera jamesonii* plants infected with nematodes^35^. The present work also reveals a further increase in activity of all the enzymes in *M. incognita* infested *S. lycopersicum* plants after treatment with biometabolites produced by *S. hydrogenans* in the form of culture cells, supernatant and extract. A rise in the activities of SOD, PPO, CAT and APOX was also found in *P. aeruginosa* and *B. gladioli* treated nematode infected tomato plants^34^. Our results are in accordance with the findings of Gupta et al.^36^. They stated an increment in the activity of SOD in microbe inoculated nematode infected *Withania somnifera* plants. Similar incrementation in the activity of PPO was observed in nematode infected tomato plants that were inoculated with *Streptomyces* sp.^37^. The main attribute behind this upregulation after amendment of biometabolites produced by *S. hydrogenans* strain DH-16 might be because of increased protein levels in treated plants in comparison to untreated nematode infected plants.

Along with enzymatic antioxidants, non-enzymatic antioxidants like tocopherol and glutathione content have also been found to be elevated in case of infected plants. Similar findings were reported previously in case of papaya, grapevine, eggplant and tomato plants grown under nematode stress^11,38,39^. This elevation might be because of ROS scavenging potential of non-enzymatic antioxidants^38,40^. Our results also depict further elevation in the levels of tocopherol and glutathione in case of biometabolites supplemented nematode infected tomato plants. Similar type of results were reported previously in case of microbial strain supplemented *Bacopa monneri* plants by Gupta et al.^33^. Our results also find co-occurrence with our previous findings^23^. According to literature, treatment of biometabolites produced by microbial strains elevated the content of tocopherol and glutathione in tomato plants. Further, this rise is attributed to the fact that these antioxidants, activated after the treatment with micro-organisms starts acting as ROS scavengers or redox buffers^41,42^.

Enhancement in the content of various phenolic compounds like total phenols, flavonoids and anthocyanin have also been found in the present study. Previous studies also state that increment in phenolic content is related to the alleviation of stress caused by pathogens^43^. The level of phenolic compound has been found to increase further after the supplementation of *Streptomyces* sp. generated biometabolites. The reason behind the elevation might be the role of biometabolites produced by microbes in mitigating stress generated in plants because of nematode infection, as also depicted by reduced number of galls. Our results are in similar lines with the findings of Khanna et al.^34^, who also reported an increase in phenolic compounds in microbes amended nematode stressed plants. Additionally, supplementation of *Chitinophillus* sp., *Streptomyces* sp. and *Cellulosimicrobium* sp. to *W. somnifera* resulted in enhanced levels of total phenols and flavonoids. The possible reason behind the enhancement might be the chelating potential of phenolic compounds against biotic stressors. Furthermore, the generation of these compounds also restricts the migration of nematodes. All these findings demonstrate the bioefficacy of metabolites produced by *Streptomyces* sp. strain DH-16 against *M. incognita* infected tomato plants by modulating the antioxidative defense system of the host plant.

## Methods

### Microbial culture

*S. hydrogenans* strain DH-16 (JX123130) was used as a biocontrol agent in the present study and was procured from the Microbiology Department, GNDU, Amritsar, Punjab^44^. The pure culture was streaked on SCNA (starch casein nitrate agar) plates and was then incubated at 28 ℃ for 4 days and was kept in reserve at 4 ℃ for further experimental work.

### Bioactive metabolites production

The yield of secondary metabolites was carried out by inoculating three stubs (6 mm) of microbial culture from fully grown plate to starch casein nitrate media (50 ml) followed by its agitation at 180 rpm and 28 ℃ in a shaker incubator for 72 h^44^. After incubation, the cells were separated from broth using filtration and then both culture cells and sterile cell free filtrate (supernatant) were used for further experimentation. Furthermore, active metabolites present in the cell free filtrate were extracted using ethyl acetate and were then concentrated under vacuum with the help of rotary evaporator. The brown-colored residue thus formed was re-dissolved in 0.5% DMSO (dimethyl sulfoxide) solution and was then stored at 4℃ for further experimentation.

### *Meloidogyne incognita* culture

*M. incognita* culture was obtained from infected roots of brinjal and tomato plants. The culture was screened and identified by studying the perennial pattern of females and then the pure culture was grown in the green house of the department. The egg masses from infected roots were collected and were washed with 1.5% sodium hypochlorite solution followed by subsequent washing in distilled water. The extracted egg masses were then placed in distilled water and were allowed to hatch at 27 ℃, followed by collection of infective second stage juveniles (J_2_) which were used for experimental studies.

### Plant material and treatments

Certified seeds of susceptible variety of *S. lycopersicum* var. Pusa Ruby were purchased from the market and were surface sterilized by dipping them in 1.5% sodium hypochlorite solution for 1 min, followed by subsequent washing in distilled water. The disinfected seeds were then sown in autoclaved soil and then the seedlings with true leaf stage were transferred into earthen pots containing autoclaved soil. After 2–3 days of transplantation, plants were subjected to different treatments which were C (Control, 0.5% DMSO), NI (Nematode infested with 2J_2_s/g of soil), CC (1 × 10^6^ Culture cells), CC + NI (1 × 10^6^ Culture cells + 2J_2_s/g of soil), S (supernatant), S + NI (supernatant + 2J_2_s/g of soil) and E (extract), E + NI (extract + 2J_2_s/g of soil). There were five replicates of each treatment and the experiment was terminated after 60 days.

### Growth parameters

Various growth parameters like height of plant (root and shoot length) and fresh biomass (root and shoot weight) were assessed. Further, number of galls per plant were also recorded in differently treated plants to check nematode infection in plants.

### Photosynthetic pigments

For the estimation of photosynthetic pigments (chl ‘a’; chl ‘b’; total chl and carotenoid content) homogenate was prepared by crushing 1 g of fresh leaf sample in 4 ml 80% acetone solution, which was then subjected to centrifugation for 20 min at 10,000 rpm and 4 ℃ . The debris free filtrate thus formed was further used for measuring its absorbance at wavelengths 645 and 663 nm for chlorophyll content and 480 and 510 nm for carotenoid content using spectrophotometer^45,46^.

### Gaseous exchange parameters

Various gaseous exchange parameters like Pn (Net photosynthetic rate), E (Transpiration rate), Gs (Stomatal conductance) and Ci (Intercellular CO_2_ rate) were assessed in 60 days old tomato plants. The assessment was done during sunny day with the help of Portable Photosynthesis Measuring System Unit (Li COR-6400, LiCOR Instruments, Lincoln, NE, USA).

### Oxidative stress markers

#### Malondialdehyde (MDA) content

1 g of fresh leaf sample was crushed in 0.1% Trichloroacetic acid (TCA, 5 ml) for the production of homogenate, followed by its centrifugation at 5000 rpm at 4 ℃. The supernatant thus formed was collected and mixed with 20% TCA containing 0.5% Thio barbituric acid (TBA) and the reaction mixture was then subjected to heating in water bath at 95 ℃ for 30 min followed by its cooling in ice cold conditions. Afterwards, the supernatant was separated and its absorbance was recorded at 532 nm^47^.

#### Hydrogen peroxide (H_2_O_2_) content

1 g of fresh leaf sample was crushed in 1% TCA (6 ml) and was then subjected to centrifugation at 12,000 rcf for 15 min. The supernatant thus formed was collected and then 0.5 ml of it was mixed with 1 ml potassium iodide and 1 ml Potassium phosphate buffer of pH 7. The optical density of final reaction mixture was then recorded at 390 nm using Genesys 20 UV visible spectrophotometer. A standard curve was made using H_2_O_2_ for further evaluation^48^.

### Antioxidative enzymes

For assessing specific activity of antioxidative enzymes, protein content was required. So, in order to evaluate the protein content as well as antioxidative enzymes, 1 g of fresh leaf sample was homogenised in 3 ml of 0.1 M Potassium phosphate buffer (pH 7.0). The homogenate thus generated was subjected to centrifugation for 20 min at 13,000 rpm. The protein content was assessed as per Lowry method^49^ and Bovine Serum Albumen (BSA) was used as the standard for estimation. The specific activity of various antioxidative enzymes like Glutathione-S-Transferase (GST) ; guaiacol peroxidise (GuPOX)); ascorbate peroxidase (APOX); polyphenol oxidase (PPO) ; catalase (CAT) ; and superoxide dismutase (SOD) were also assessed].

#### GST (EC. 2.5.1.18)

Specific activity of GST was examined by using the protocol given by Habig and Jakoby^50^. The reaction was started by mixing potassium phosphate buffer (20 mM, pH 7.5); reduced glutathione (20 mM); 1-chloro 2,4-dinitrobenzene (Cdnb, 20 mM) and plant extract. The absorbance was recorded at 340 nm.

#### GuPOX (EC.1.11.1.7)

Specific activity of GuPOX was assessed as per the methodology proposed by Putter^51^. The components of reaction mixture include potassium phosphate buffer (20 mM; 7.0); freshly prepared guaiacol solution (10 mM); hydrogen peroxide (124 mM) and plant extract. The increment in optical density was noted at 436 nm.APOX (EC.1.11.1.11).

Specific activity of APOX was calculated by the methodology given by Nakano and Asada^52^. The reaction mixture consists of ascorbate (0.5 mM); potassium phosphate buffer (50 mM; 7.0); hydrogen peroxide (2 mM) and plant extract. The absorbance was recorded at 290 nm.

#### PPO (EC.1.14.18.1)

The specific activity of PPO was determined using the protocol given by Esterbauer et al.^53^. The components of reaction mixture include potassium phosphate buffer (0.1 M; pH 7.0); catechol (0.1 M) and enzyme extract. The absorbance of reaction mixture was recorded at wavelength 412 nm.

#### CAT (EC.1.11.1.6)

The specific activity of CAT was determined using the standard protocol given by Aebi^54^. The components of reaction mixture involve potassium phosphate buffer (50 mM, pH 7.0); hydrogen peroxide (20 mM) and plant extract. Dismutation of hydrogen peroxide resulted in decrease in absorbance at 240 nm.

#### SOD (EC.1.15.1.1)

The specific activity of SOD was examined by the procedure given by Kono^55^. The components of reaction mixture include sodium carbonate buffer (50 mM); hydroxylamine hydrochloride (1 mM; pH 6.0); Triton-X 100 (0.03%); nitro blue tertrazolium (NBT, 24 µM), ethylenediaminetetraacetic acid (EDTA) and plant extract. The enhancement in optical density was recorded at 540 nm.

### Non-enzymatic antioxidants

Tocopherol and total glutathione content were also assessed in the current study. For assessing both the parameters the homogenate was prepared by homogenising 1 g of fresh leaf sample in 50 mM Tris buffer (10 ml; pH 10) and was then subjected to centrifugation for 15 min at 12,000 rcf, followed by collection of supernatant, which was then used for evaluating Tocopherol and total glutathione content.

#### Tocopherol content

The supernatant (0.5 ml) collected was mixed with double distilled water (0.5 ml) and absolute ethanol (0.5 ml), followed by vigorous shaking. Xylene (0.5 ml) was added to the reaction mixture and was then subjected to centrifugation for 10 min at 3000 rcf, that results in the formation of two layers. The upper xylene layer was collected and 2,4,6-tripyridyl-s-triazine (TPTZ) was added to it. The absorbance of reaction mixture thus formed was recorded at 600 nm^56^.

#### Total glutathione content

The supernatant (0.1 ml) collected was mixed with methanol (4 ml) and 5,5′-dithiobis-(2-nitrobenzoic acid) (50 µl) and was incubated for 15 min at room temperature, followed by centrifugation for 15 min at 3000 rcf. The optical density of resultant mixture thus formed was recorded at 412 nm^57^.

### Phenolic compounds

#### Flavonoid content

The homogenate for the evaluation of flavonoid content was prepared by crushing fresh leaf sample in 80% ethanol solution, followed by centrifugation for 20 min at 10,000 rpm. The supernatant (100 µl) thus formed was mixed with methanol (2.9 ml), aluminium chloride (100 µl), 5% sodium potassium tartarate (100 µl) and distilled water (500 µl). The reaction mixture thus formed was subjected to vigorous shaking and was incubated for 30 min. The optical density of final mixture was recorded at 415 nm wavelength using spectrophotometer^58^.

#### Anthocyanin content

For estimation of anthocyanin content, fresh plant sample was crushed in acidified methanol, containing (V/V/V) methanol, distilled water and hydrochloric acid (79:20:1). The homogenate formed was incubated overnight, followed by centrifugation at 10,000 rcf for 15 min. The optical density of supernatant was then recorded at 530 and 657 nm^59^.

#### Total Phenolic content

Homogenate for determination of total phenolic content was prepared using 80% methanol, followed by centrifugation at 10,000 rpm for 20 min. The supernatant was separated from pellet and 100 µl supernatant was mixed with 2.9 ml distilled water, 500 µl FC reagent:water (1:1) and 2 ml of 20% sodium carbonate, followed by vigorous shaking. Then the absorbance of final reaction mixture was recorded at 760 nm^60^.

### Microscopy

#### Cell viability

For assessing cell viability, root tips of fresh *S. lycopersicum* plants were dipped in 50 µM Propidium iodide (PI) solution and then were incubated at room temperature for 10–15 min. The extra PI was removed with the help of double distilled water. The stained root tips were then mounted on slide and was observed under Nikon AIR Confocal Laser Scanning Microscope, under the excitation and emission wavelength of 543 and 617 nm respectively.

#### Nuclear damage

For assessing nuclear damage, fresh root tips were dipped in 4,6-diamino-2-phenylindole (DAPI) and were incubated for 15 min in dark followed by washing in deionized water. Root tips were mounted on slide and was observed under Nikon AIR Confocal Laser Scanning Microscope, at an excitation and emission wavelength of 358 and 461 nm respectively.

#### Hydrogen peroxide localization

For assessing hydrogen peroxide localization, fresh root tips of plants were immersed in Dichlorofluorescein-diacetate (DCF-DA) for 20 min under dark conditions followed by washing in deionized water. Samples were then mounted on glass slide and were observed under Nikon AIR Confocal Laser Scanning Microscope, at an excitation and emission wavelength of 485 and 535 nm respectively.

### Statistical analysis

All the calculations (one-way Anova and Tukey’s test) were performed by using SPSS software and by self-coded office excel software.

### Ethical statement

Experimental research on plant complies with relevant institutional, national and international guidelines and legislation.

## Conclusion

*M. incognita* infestation in *S. lycopersicum* plants has resulted in impaired growth attributes that is also related to enhanced production of ROS (oxidative stress markers) and modulation of antioxidative defense system of host plant. However, the destruction caused by RKN infestation can be alleviated by the application of microbial strains that exhibit nematicidal properties. Further, the microbial strains are also involved in supressing the level of stress markers and thus helps in maintaining the normal physiology of plants even under stressful conditions by upregulating the level of various enzymatic and non-enzymatic antioxidants in plants. Thus, all these finding prove that *S. hydrogenans* strain DH-16 possess the exquisite potential for plant growth promotion and mitigation of nematode stress in plants. However, further research is required to understand the molecular mechanism involved in mitigation of nematode stress in plants after microbial supplementation.

## Supplementary Information

Supplementary Information.

## Data Availability

The data that support the findings of this study will be made available from the corresponding author upon request for replication purposes.
