# Supplementary material for: Streptomyces hydrogenans strain DH-16 alleviates negative impact of Meloidogyne incognita stress by modifying physio-biochemical attributes in Solanum lycopersicum plants
Source: Sci Rep. 2022 Sep 8;12:15214. doi: 10.1038/s41598-022-19636-0 (PMC9458671; doi:10.1038/s41598-022-19636-0)
Supplement: Supplementary file 1 — Supplementary Information. [file 41598_2022_19636_MOESM1_ESM.docx]

***Streptomyces hydrogenans* strain DH-16 Alleviates Negative Impact of *Meloidogyne incognita* Stress by Modifying Physio-biochemical Attributes in *Solanum lycopersicum* Plants.**

Nandni Sharma^1^, Rajesh Kumari Manhas^2^, Puja Ohri^1*^

^1^ Department of Zoology, Guru Nanak Dev University, Amritsar, Punjab 143005, India; [sharmanandni1303@gmail.com](mailto:sharmanandni1303@gmail.com); ohri_puja_11@rediffmail.com

^2^ Department of Microbiology, Guru Nanak Dev University, Amritsar, Punjab 143005, India; [rk.manhas@rediffmail.com](mailto:rk.manhas@rediffmail.com)

**Table S1.** Effect of supplementation of biometabolites produced by *S. hydrogenans* strain DH-16 on photosynthetic pigments of *S. lycopersicum* plants 60-days after nematode inoculation.

| **TREATMENTS** | **Photosynthetic pigments (Mean±S.E.)** | | | |
| --- | --- | --- | --- | --- |
|  | **Chlorophyll ‘a’**  **(mg/g FW)** | **Chlorophyll ‘b’**  **(mg/g FW)** | **Total Chlorophyll content**  **(mg/g FW)** | **Total carotenoid content**  **(mg/g FW)** |
| **C** | 2.1499±0.21613^ab^ | 1.1707±0.13370^ab^ | 3.2842±0.13295^ab^ | 0.3383±0.00532^ab^ |
| **NI** | 0.0890±0.00470^a^ | 1.8420±0.5021^bc^ | 1.8961±0.05135^a^ | 0.2784±0.00833^a^ |
| **CC** | 3.0115±0.25049^b^ | 1.6473±0.06776^bc^ | 4.6075±0.30770^b^ | 0.5462±0.02705^c^ |
| **CC+NI** | 1.7650±0.61660^ab^ | 2.0846±0.13099^c^ | 3.7987±0.50715^b^ | 0.4396±0.05902^bc^ |
| **S** | 3.5917±0.59003^b^ | 0.7791±0.19365^a^ | 4.3317±0.40284^b^ | 0.3295±0.02292^ab^ |
| **S+NI** | 1.7227±0.11959^ab^ | 2.1810±0.05128^c^ | 3.8514±0.16688^b^ | 0.3944±0.00970^ab^ |
| **E** | 2.6060±0.14926^b^ | 2.3187±0.14386^c^ | 4.8637±0.27920^b^ | 0.3668±0.01689^ab^ |
| **E+NI** | 2.9510±0.79571^b^ | 0.6623±0.35698^a^ | 3.5808±0.45017^b^ | 0.2820±0.00376^a^ |
| **F-value** | 6.209** | 14.312** | 8.140** | 12.073** |

** indicates significance at p ≤ 0.01. Different letters a, b, c represents significant difference among treatments. Different treatments in table are represented as C (control), NI (nematode inoculated), CC (culture cells), CC + NI (culture cells + nematode inoculated), S (supernatant), S + NI (supernatant + nematode inoculated), E (extract) and E + NI (extract + nematode inoculated).

**Table S2**: Effect of supplementation of biometabolites produced by *S. hydrogenans* strain DH-16 on gas exchange parameters of *S. lycopersicum* plants 60-days after nematode inoculation.

| **TREATMENTS** | **Gas exchange parameters (Mean±S.E.)** | | | |
| --- | --- | --- | --- | --- |
|  | **Photosynthetic rate**  **(µm CO_2_ m^-2^S^-1)^** | **Stomatal conductance**  **(mmole H_2_O m^-2^S^-1^)** | **Carbon intake (µmole CO_2_ mole^-1^)** | **Transpiration rate (mmole H_2_O m^-2^S^-1^)** |
| **C** | 8.8233±0.23104^c^ | 0.2890±0.01562^cd^ | 349±3.71184 | 6.3100±0.18735^bcd^ |
| **NI** | 2.3667±0.50167^a^ | 0.0477±0.01647^a^ | 313±12.76715 | 1.7187±0.57367^a^ |
| **CC** | 9.4167±0.39193^c^ | 0.3880±0.06243^d^ | 348±6.80686 | 8.9167±0.57202^d^ |
| **CC+NI** | 7.3867±0.52046^bc^ | 0.2387±0.01161^bc^ | 353±5.23874 | 8.7367±1.35009^cd^ |
| **S** | 8.8733±0.32256^c^ | 0.3050±0.02101^cd^ | 325±37.57753 | 5.8433±0.39960^bc^ |
| **S+NI** | 6.8267±0.93727^bc^ | 0.1727±0.02852^abc^ | 316±3.38296 | 3.6100±0.15716^ab^ |
| **E** | 6.9133±0.67681^bc^ | 0.2217±0.02839^bc^ | 340±12.12436 | 5.9100±0.34356^bc^ |
| **E+NI** | 5.7133±0.35177^b^ | 0.1314±0.02134^ab^ | 337±7.79423 | 5.2300±0.06429^b^ |
| **F-value** | 17.922** | 12.972** | Ns | 16.364** |

** indicates significance at p ≤ 0.01; (ns) indicates non-significant difference. Different letters a, b, c, d represents significant difference. Different treatments in table are represented as C (control), NI (nematode inoculated), CC (culture cells), CC + NI (culture cells + nematode inoculated), S (supernatant), S + NI (supernatant + nematode inoculated), E (extract) and E + NI (extract + nematode inoculated).

**Table S3**: Effect of supplementation of biometabolites produced by *S. hydrogenans* strain DH-16 on oxidative stress markers of *S. lycopersicum* plants 60-days after nematode inoculation.

| **TREATMENTS** | **Oxidative stress markers (Mean±S.E.)** | |
| --- | --- | --- |
|  | **MDA content (mmol/g FW)** | **Hydrogen peroxide content (µmol/g FW)** |
| **C** | 6.8946±0.30108^a^ | 0.2068±0.02546^ab^ |
| **NI** | 9.2882±0.30888^d^ | 0.4941±0.01401^c^ |
| **CC** | 7.1054±0.14360^ab^ | 0.1934±0.04096^ab^ |
| **CC+NI** | 8.6559±0.24644^cd^ | 0.1941±0.01966^ab^ |
| **S** | 7.0151±0.43422^ab^ | 0.1318±0.00754^a^ |
| **S+NI** | 8.3097±0.35272^bcd^ | 0.2448±0.03464^ab^ |
| **E** | 7.1204±0.05428^ab^ | 0.2241±0.04153^ab^ |
| **E+NI** | 7.4516±0.28681^abc^ | 0.3168±0.03012^b^ |
| **F-value** | 9.735** | 14.613** |

** indicates significance at p ≤ 0.01. Different letters a, b, c, d represents significant difference. Different treatments in table are represented as C (control), NI (nematode inoculated), CC (culture cells), CC + NI (culture cells + nematode inoculated), S (supernatant), S + NI (supernatant + nematode inoculated), E (extract) and E + NI (extract + nematode inoculated).

**Table S4**: Effect of supplementation of biometabolites produced by *S. hydrogenans* strain DH-16 on non-enzymatic antioxidants of *S. lycopersicum* plants 60-days after nematode inoculation.

| **TREATMENTS** | **Non-enzymatic antixoidants (Mean±S.E.)** | |
| --- | --- | --- |
|  | **Total Glutathione content**  **(mg/g FW)** | **Tocopherol content**  **(mg/g FW)** |
| **C** | 0.1070±0.00788^a^ | 0.1561±0.03885^a^ |
| **NI** | 0.2386±0.00321^b^ | 0.3248±0.01856^b^ |
| **CC** | 0.1486±0.02843^a^ | 0.4541±0.01179^bcd^ |
| **CC+NI** | 0.2813±0.01656^bc^ | 0.5165±0.01374^d^ |
| **S** | 0.1703±0.00601^a^ | 0.3591±0.01234^bc^ |
| **S+NI** | 0.3073±0.01593^c^ | 0.5021±0.02718^cd^ |
| **E** | 0.1573±0.00133^a^ | 0.4338±0.04883^bcd^ |
| **E+NI** | 0.2883±0.01040^bc^ | 0.5038±0.03823^d^ |
| **F-value** | 29.546** | 17.396** |

** indicates significance at p ≤ 0.01. Different letters a, b, c, d represents significant difference among treatments. Different treatments in table are represented as C (control), NI (nematode inoculated), CC (culture cells), CC + NI (culture cells + nematode inoculated), S (supernatant), S + NI (supernatant + nematode inoculated), E (extract) and E + NI (extract + nematode inoculated).

Table S5: Effect of supplementation of biometabolites produced by *S. hydrogenans* strain DH-16 on phenolic compounds of *S. lycopersicum* plants 60-days after nematode inoculation.

| **TREATMENTS** | **Phenolic compounds (Mean±S.E.)** | | |
| --- | --- | --- | --- |
|  | **Anthocyanin content (mg/g FW)** | **Flavonoid**  **content (mg/g FW)** | **Total phenolic content (mg/g FW)** |
| **C** | 1.5639±0.3555^ab^ | 0.0973±0.00521^ab^ | 0.1833±0.02242^ab^ |
| **NI** | 1.5978±0.12256^ab^ | 0.0731±0.00997^a^ | 0.0499±0.02793^a^ |
| **CC** | 0.9339±0.22395^a^ | 0.1036±0.01330^ab^ | 0.2106±0.01330^b^ |
| **CC+NI** | 1.6932±0.13027^ab^ | 0.0896±0.00481^ab^ | 0.1826±0.04044^ab^ |
| **S** | 1.4187±0.26882^ab^ | 0.913±0.00240^ab^ | 0.2213±0.00639^b^ |
| **S+NI** | 1.2682±0.19656^ab^ | 0.1190±0.01365^b^ | 0.1689±0.02862^ab^ |
| **E** | 1.2751±0.22273^ab^ | 0.1143±0.00984^ab^ | 0.2313±0.05783^b^ |
| **E+NI** | 2.1707±0.16092^a^ | 0.1123±0.00657^ab^ | 0.2016±0.01810^b^ |
| **F-value** | 2.695* | 2.855* | 3.414* |

* indicates significance at p ≤ 0.05. Different letters a, b, c, d represents significant difference among treatments and same letters represents no significant difference. Different treatments in table are represented as C (control), NI (nematode inoculated), CC (culture cells), CC + NI (culture cells + nematode inoculated), S (supernatant), S + NI (supernatant + nematode inoculated), E (extract) and E + NI (extract + nematode inoculated).
